# Validation of the F-POD—A fully automated cetacean monitoring system

**DOI:** 10.1371/journal.pone.0293402

**Published:** 2023-11-17

**Authors:** Julia Ivanchikova, Nicholas Tregenza

**Affiliations:** 1 Schmalhausen Institute of Zoology of National Academy of Sciences of Ukraine, Kyiv, Ukraine; 2 Sea Mammal Research Unit, Scottish Oceans Institute, University of St Andrews, Scotland, United Kingdom; 3 Chelonia Limited, Mousehole, Cornwall, United Kingdom; MARE – Marine and Environmental Sciences Centre, PORTUGAL

## Abstract

The F-POD, an echolocation-click logging device, is commonly used for passive acoustic monitoring of cetaceans. This paper presents the first assessment of the error-rate of fully automated analysis by this system, a description of the F-POD hardware, and a description of the KERNO-F v1.0 classifier which identifies click trains. Since 2020, twenty F-POD loggers have been used in the BlackCeTrends project by research teams from Bulgaria, Georgia, Romania, Türkiye, and Ukraine with the aim of investigating trends of relative abundance in populations of cetaceans of the Black Sea. Acoustic data from this project analysed here comprises 9 billion raw data clicks in total, of which 297 million were classified by KERNO-F as Narrow Band High Frequency (NBHF) clicks (harbour porpoise clicks) and 91 million as dolphin clicks. Such data volumes require a reliable automated system of analysis, which we describe. A total of 16,805 Detection Positive Minutes (DPM) were individually inspected and assessed by a visual check of click train characteristics in each DPM. To assess the overall error rate in each species group we investigated 2,000 DPM classified as having NBHF clicks and 2,000 DPM classified as having dolphin clicks. The fraction of NBHF DPM containing misclassified NBHF trains was less than 0.1% and for dolphins the corresponding error-rate was 0.97%. For both species groups (harbour porpoises and dolphins), these error-rates are acceptable for further study of cetaceans in the Black Sea using the automated classification without further editing of the data. The main sources of errors were 0.17% of boat sonar DPMs misclassified as harbour porpoises, and 0.14% of harbour porpoise DPMs misclassified as dolphins. The potential to estimate the rate at which these sources generate errors makes possible a new predictive approach to overall error estimation.

## Introduction

The use of autonomous instruments to monitor wild animals is becoming increasingly widespread and important but raises problems of interpreting the data collected and verifying that interpretation [1–3] Photographic, video [4] and acoustic [5, 6] data sets can be massive and inspection by human analysts may become a significant component of a project, bringing both high costs and unwelcome subjectivity. At the same time there has been rapid progress in developing automated digital classifiers that aim to equal or outdo the human analyst.

Odontocetes produce a wide variety of sounds for different purposes [7]. Clicks are rapid high-frequency discrete sounds made by toothed whales for communication and used for echolocation to find prey and navigate [8]. Clicks are usually produced in sequences termed trains, and static acoustic instruments capture fragments of these trains as the cetacean’s sound beam sweeps across the hydrophone. The characteristics of click trains offer more valuable information for classification purposes in comparison to analysing individual clicks separately.

PODs (POrpoise Detectors) [9] are a family of self-contained click loggers widely used for passive acoustic monitoring of toothed whales in the last two decades [10–20]. With the introduction of the T-POD (Timing POrpoise Detector) this type of logger “has become a standard tool in environmental impact assessments and monitoring programmes” [21]. Subsequently C-PODs (Cetacean POrpoise Detectors) were used to detect trends in the declining population of the vaquita in the acoustically difficult context of the Upper Gulf of California [17, 18]. C-PODs were used to estimate the abundance of the critically endangered Baltic Sea harbour porpoises [20] in the Static Acoustic Monitoring of the Baltic Sea Harbour Porpoise (SAMBAH) project [22]. PODs were used for detection of trends in the harbour porpoise population in the Minas passage, Canada [23]. PODs have been used to investigate behaviour changes of porpoises in response to man-made structures such as oil and gas platforms [24] and to assess the response of dolphins and porpoises to pingers [25].

PODs aim to record and identify odontocete click trains. The data from PODs have been commonly used to make inferences about the occurrence [16, 26–29] and behaviour [30–36] of various species of cetaceans using the results of the automated post-processing. This process was the KERNO classifier for the C-POD logger, with some additional encounter classifiers, such the ‘Hel1’ classifier [9] used in the SAMBAH project on a species at very low density where false positives are a major issue. It is essential that the output of this process has a low error rate [15, 20] if it is to be used without editing.

The key threshold in classifier development is reaching a performance level where data can be used without human editing of the results, and here we assess this level achieved by the KERNO-F classifier operating on data from the F-POD (Full waveform capture POD) instruments in the BlackCeTrends project. Technical aspects of the classifier and instruments are in S1 File.

Here we describe assessments of errors in the output of the F-POD hardware combined with the custom post-processing by the FPOD.exe software. We focus primarily on false positive errors in classification by Version 1.0 of the newly developed KERNO-F classifier and present its first assessment based on a large field data set, including records of porpoises, dolphins, sonars and other sources of high frequency sounds.

In this paper we address:

1. Validation of the project as a whole– to estimate the incidence of errors.
2. Validation of each file–this is required to assess the conditions of recording of each data file.
3. Assessment of the source of errors.

## Materials and methods

### Background, rationale, and principles of error rate assessment

Error rates may vary between species and locations because some click types are more distinctive than others, and some locations have more potential sources of clicks or click trains that may be confused with the target species.

In the Black Sea all three species of cetaceans are odontocetes and produce click trains. Black Sea harbour porpoises (*Phocoena phocoena relicta)* [37, 38] produce Narrow-Band High Frequency (NBHF) click trains [39] which can be discriminated from the mostly Broad-Band Transient click trains of delphinids, which in the Black Sea are the common dolphin (*Delphinus delphis ponticus)* [40] and the bottlenose dolphin (*Tursiops truncatus ponticus)* [41]. When boat sonar pulses are detected at a distance from their origin, they may resemble harbour porpoise clicks (NBHF clicks). Alternatively, these sonar pulses can resemble dolphin clicks if they have been fragmented into shorter clicks during propagation.

The data collected by F-PODs comprise a list of clicks that are selected by real-time processing of the incoming stream of digitised sound. The F-POD has a fixed sampling rate of 1 million 10 bit-samples per second. This is upsampled in real time within the POD by zero-stuffing to 4 million samples per second, followed by Gaussian low-pass filtering to give 250ns resolution in the timing of individual cycle peaks and inter-peak-intervals (IPIs). Time domain analysis is then used to select clicks for storage of a set of click features. These features include the position of the loudest cycle in the click, the range of inter-peak-intervals (IPIs), the number of reversals in the trend of cycle amplitudes, the IPIs and cycle amplitudes around the loudest cycle, the last IPI value, the IPI before the click start and the time of the click (to 5 μs resolution). These click features are stored in 16 bytes for each selected click, which may be between 1 and 255 cycles long. This data compression typically allows 1 year of data to be stored in much less than 32GB.

More details of this real-time process, the data structure, the code to unpack it, and an outline of the KERNO-F algorithm are given in S1 File.

Data from earlier versions of the POD have been evaluated in several studies by comparing the results from the POD with visual observations of cetaceans in the vicinity of the POD. Philpott [42] found that 82% of visual observations of groups of bottlenose dolphins were logged by a POD, while some groups logged by a POD were not seen. Good levels of correlation of POD data with visual observation has also been found in other studies [14, 43–46].

The KERNO-F v1.0 classifier places possible click trains in one of four species categories: ‘NBHF’ for species producing narrow-band high frequency clicks [21, 39, 47, 48]. “Other cet” for all other odontocete species, “Sonar” for man-made sonars, and an “Unclassified” source category, which holds clicks that do not meet the minimum criteria for inclusion in any of the other 3 categories. In the Black Sea the “NBHF” category relates only to harbour porpoise and the “Other cet” category covers the two dolphin species. Here we use the terms “Porpoise” and “Dolphins” for these categories. The KERNO-F classifier also assigns a level of confidence (High, Moderate, Low, the Quality level, also known as “Q”) that each identified click train came from an actual single source of click trains rather than being a coincidental sequence of clicks that came from different sources, e.g. from multiple shrimps clicking at cetacean frequencies. Higher Q levels represent a lower false positive risk and entail reduced sensitivity.

From the structure of the classifier, it is possible to identify several factors which potentially increase error rates:

1. Sources of clicks: sediment transport such as fine sand in suspension produces huge numbers of clicks [9, 49, 50] that may by chance fall into a sequence resembling a cetacean click train. Other sources include propellers, shrimps, breaking waves, and rain.
2. Sources of click trains: boat sonars, acoustic Doppler current profilers, fish tags, acoustic modems, some weak unidentified sources, and cetaceans can all produce click trains that may be placed in the wrong species category by the classifier and may bias the classifier against identifying a click train as coming from a different species group.

These factors can lead to errors made by the KERNO-F algorithm that include: misclassification as click trains sequences that come from multiple non-cetacean sources (e.g. shrimps) that do not produce trains (false positive errors); failure to classify as click trains some trains that are present in the raw data (false negative errors); errors in classifying the train to the correct cetacean species category (false positive or negative errors); errors in identifying the click-rate of a train.

False positive errors are important to consider, particularly in studies of small and/or declining populations. The rate of true positives in the data should track the relative abundance of the population of interest. But the false positive error rate in the data may be determined by, for example, storms and waves generating surface and seabed noise, various types of seabed noise (including shrimps), the presence of non-target cetacean species and boat sonars, which will generally be independent of target population abundance. In declining populations, the proportion of false positives compared to true positives will generally increase, potentially compromising inference about population trends. This effect would be increased in very small populations.

This paper aims to estimate the false positive error rate in F-POD data collected as part of the BlackCeTrends project and assess whether these data can be used without editing.

### Acoustic deployments

**Fig 1:**
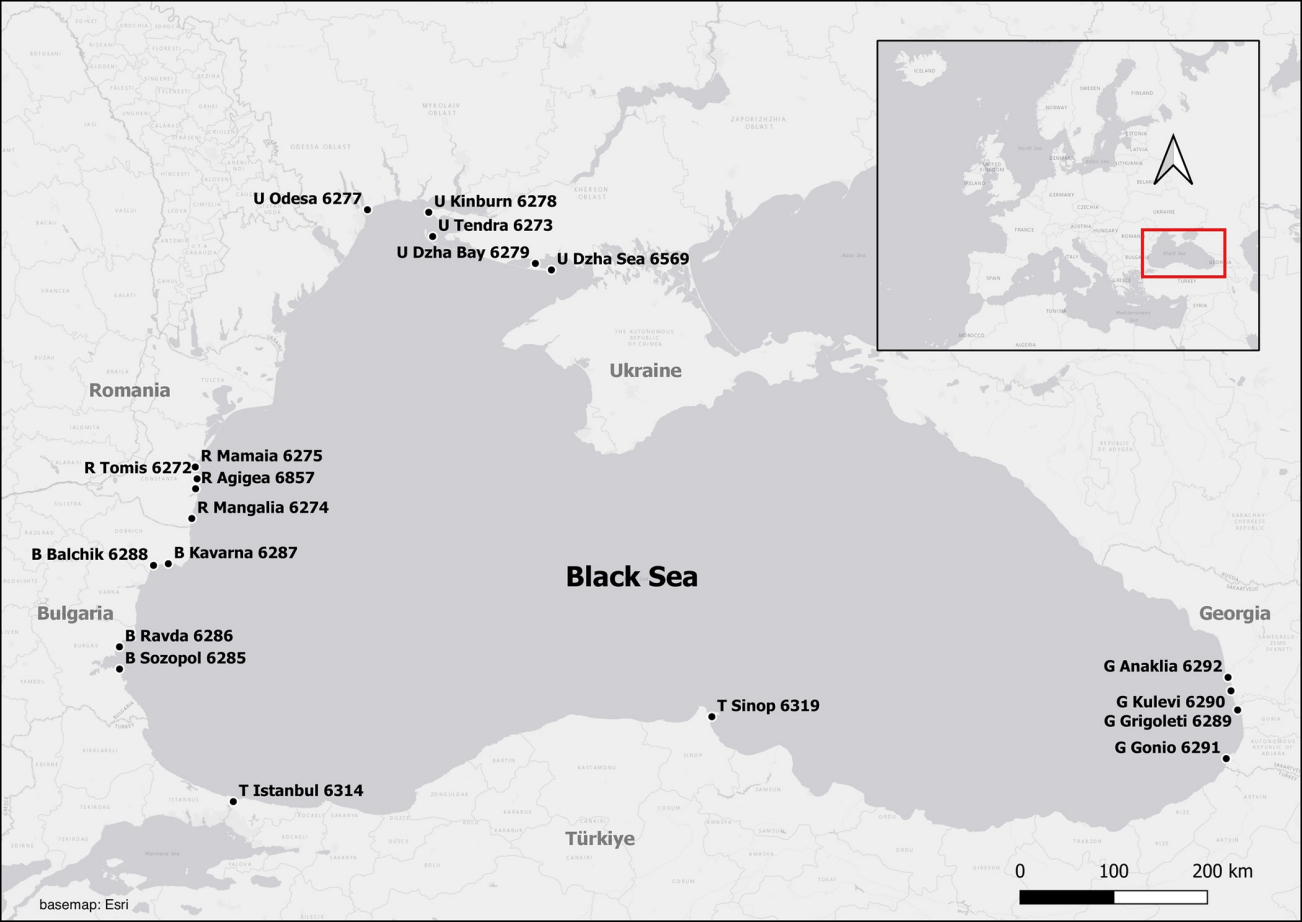
Map of the locations of F-PODs in the Black Sea. Information about locations of F-PODs, numbers, depths, and seabed type is given in Table 1 Locations in the S2 File.

Data for this study came from the international static acoustic monitoring BlackCeTrends project, which investigates the population trends and behaviour of Black Sea dolphins and harbour porpoises. The project started in September 2020, and includes five Black Sea countries: Bulgaria, Georgia, Romania, Türkiye, and Ukraine (Fig 1). The dataset comprised 87 files, covering durations from 7 to 163 logging days.

### Data processing and error evaluation

The raw data from F-PODs is stored as.FP1 files which are manually cropped to remove periods before or after immersion or affected by the presence of the servicing vessel. This produces a set of files, which were used here (see Table 2 FileList in S2 File).

Classification of click trains was made by the KERNO-F v1.0 classifier, (see further details in S1 File “Post-processing”) which takes an FP1 file and produces an FP3 file which contains only clicks found to be part of a click train. Three independent classifications were used. First, each click train is assigned to one of the four categories: “NBHF” (here Porpoise), “Other cetacean” (here Dolphins), “Sonar” and “Unclassified”. Second, the classifier places each click train in one of two classes for species identification confidence, also called “quality” (“High” or “Moderate”). Third, the classifier assigns a value for the confidence in the click rate of the train (on the scale from 0 to 15) with higher values indicating higher confidence. In this study we did not analyse click rates so all classes of click rate confidence were used. The standard click train filters for cetacean monitoring were used throughout:

1. Porpoise only or Dolphins only.
2. Quality High or Moderate (the subset of the KERNO-F output that is typically used in monitoring studies)
3. Species identification confidence High (use of only High species confidence click trains resulted in a loss of 3.2% Porpoise DPM and 2.8% Dolphin DPM).

The unit of information used here to assess false positive error rate was a Detection Positive Minute (DPM), that is a minute with detections of the species at the quality levels chosen. The study incorporated two sampling regimes.

The first sampling regime was to inspect 100 DPM for each species group in each file. The purpose of this was to identify and quantify any anomaly that may exist in a file due to malfunction of the instrument or to acoustic features of the site. To evaluate the 87 files, we chose 100 randomly selected DPM from each, using a utility in the F-POD software which reads the FP3 data file twice. In the first pass, the number of clicks that met the specified click train filters was found.

In the second pass, a starting point is randomly placed by the software within the first 1% of the set of clicks that met the specified filters and subsequent markers placed automatically at a regular spacing defined by the total number of clicks, to give evenly stratified sampling of detections. The 100 selected DPMs from each file, or all DPMs if there were fewer than 100 DPM in one file, were then examined visually. All click trains within each selected DPM were evaluated. The number of sampling points is not fixed at 100 but can be adjusted to any required whole number. If the file consisted of less then 100 DPM we evaluated all those DPMs.

The second sampling regime was implemented to generate a representative sample across the whole data set of 2000 DPM for each species group in order to give an estimate of the overall false positive error rate for each species group. This regime used the DPMs selected in the first regime as a basis. To obtain a representative sample across the whole project, we first calculated how many of the total 2000 DPMs should be evaluated in each file based on the proportion of the total DPMs that were in that file. In those files from which fewer than 100 were required we reduced proportionally the number of previously identified errors in that file. Where the number of DPMs required from a file was greater than the number already evaluated, we increased the set of DPMs to be evaluated using the procedure described above for the first sampling regime.

A DPM was considered a false positive if a single click train of the target species category (Porpoise or Dolphins) within it had been misclassified. There were on average 10 trains per minute and a minute would not cease to be detection positive if one or more was false unless all were false. Meeting this stringent criterion makes it possible in future data utilization to select either the number of clicks or DPM as an operational statistic.

**Fig 2:**
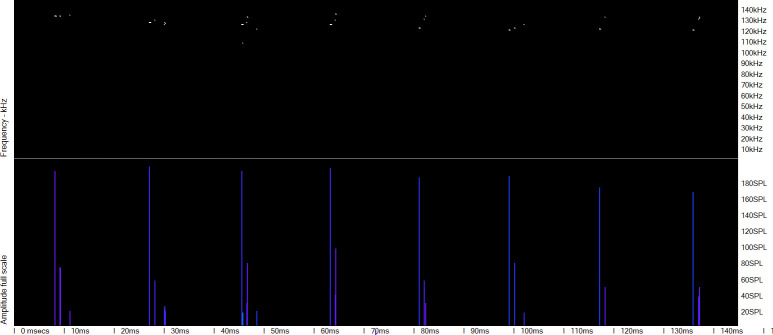
Mulitpath replicates of clicks in a porpoise click train. Frequency of clicks is shown by a spectral colour series:red = 20 kHz to violet = 160 kHz. Most of the replicate click clusters following the successive loud high frequency clicks show similar patterns of click frequency and delay to the previous cluster, and contain only frequencies within the narrow band typical of this species. This pattern is highly characteristic of Porpoise click trains.

**Fig 3:**
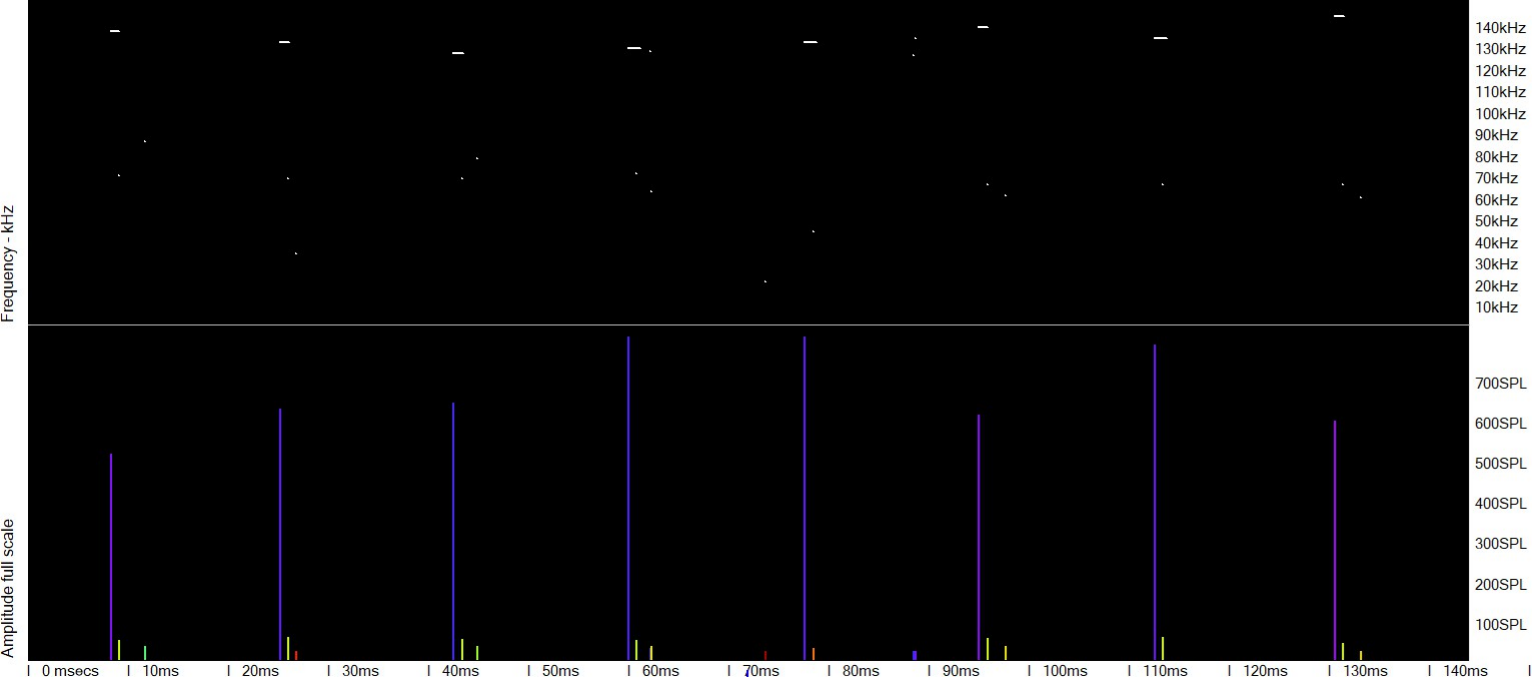
Mulitpath replicates of clicks in a dolphin click train. Frequency of clicks is shown by a spectral colour series:red = 20 kHz to violet = 160 kHz. Most of the replicate click clusters following the successive loud high frequency clicks show similar patterns of click frequency and delay to the previous cluster, and contain much lower frequencies than the initial, direct path, click. This pattern is highly characteristic of a dolphin click.

**Fig 4:**
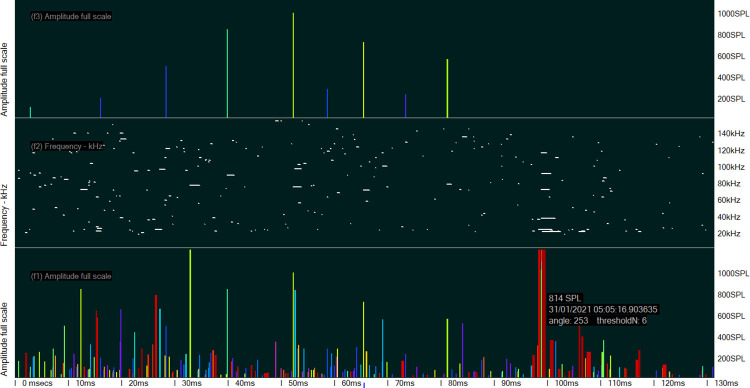
An example of a spurious click train. The two lower panels are the raw data in the FP1 file. The louder clicks that are identified as being in a train are shown in the upper panel. In the raw data, there are strongly differing sequences of clicks following these louder clicks picked out by the classifier, unlike true click trains from a cetacean.

**Fig 5:**
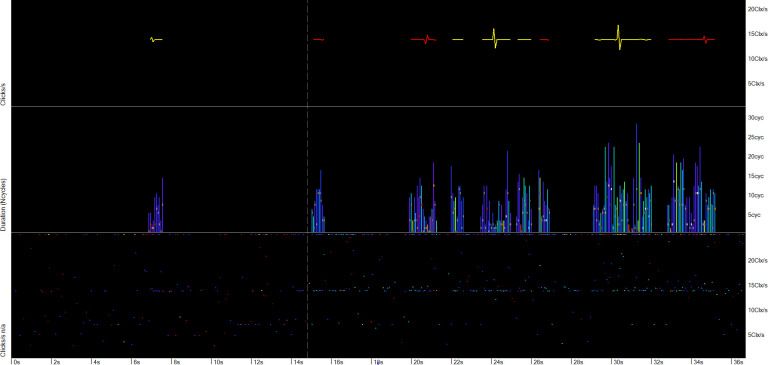
Inter-click interval of the raw click. The lower panel shows the inter-click interval of the raw clicks in the FP1 file, represented as a click rate, i.e. 1/ICI the line of constant ICIs is highly characteristic of a sonar. The middle panel shows the number of cycles in each click in identified trains, with the wavenumber of the loudest wave within the click (see S1 File, Raw data capture) marked by a small dot (lower positions are earlier in the click). The upper panel shows the click rate identified by the classifier. The lack of consistency in these values of wavenumber of the loudest wave is also highly characteristic of a sonar.

**Fig 6:**
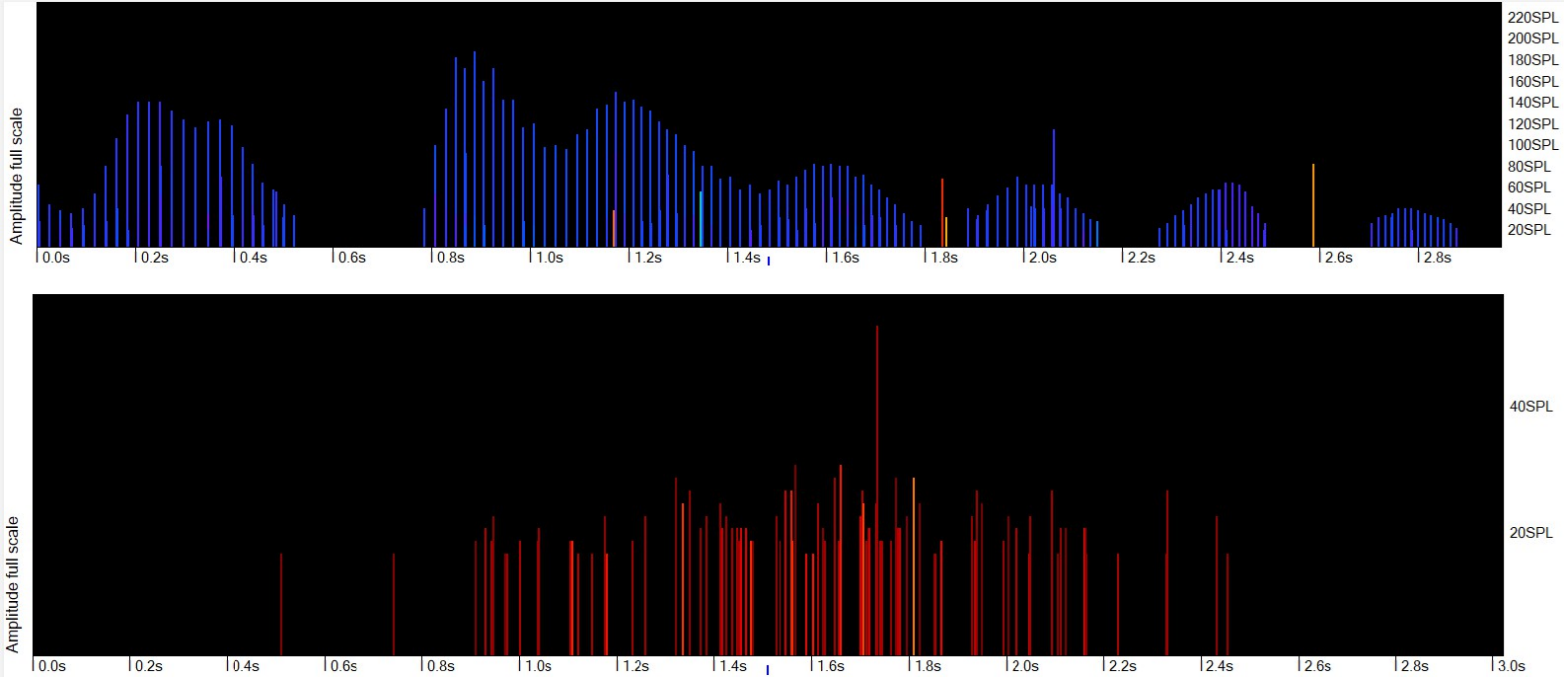
Harbour porpoise smooth amplitude profile. Upper panel: Amplitude of clicks in a porpoise click train showing the characteristic smooth amplitude profiles. Lower panel: clicks without a smooth amplitude profile which is typical of clicks from unrelated sources.

**Fig 7:**
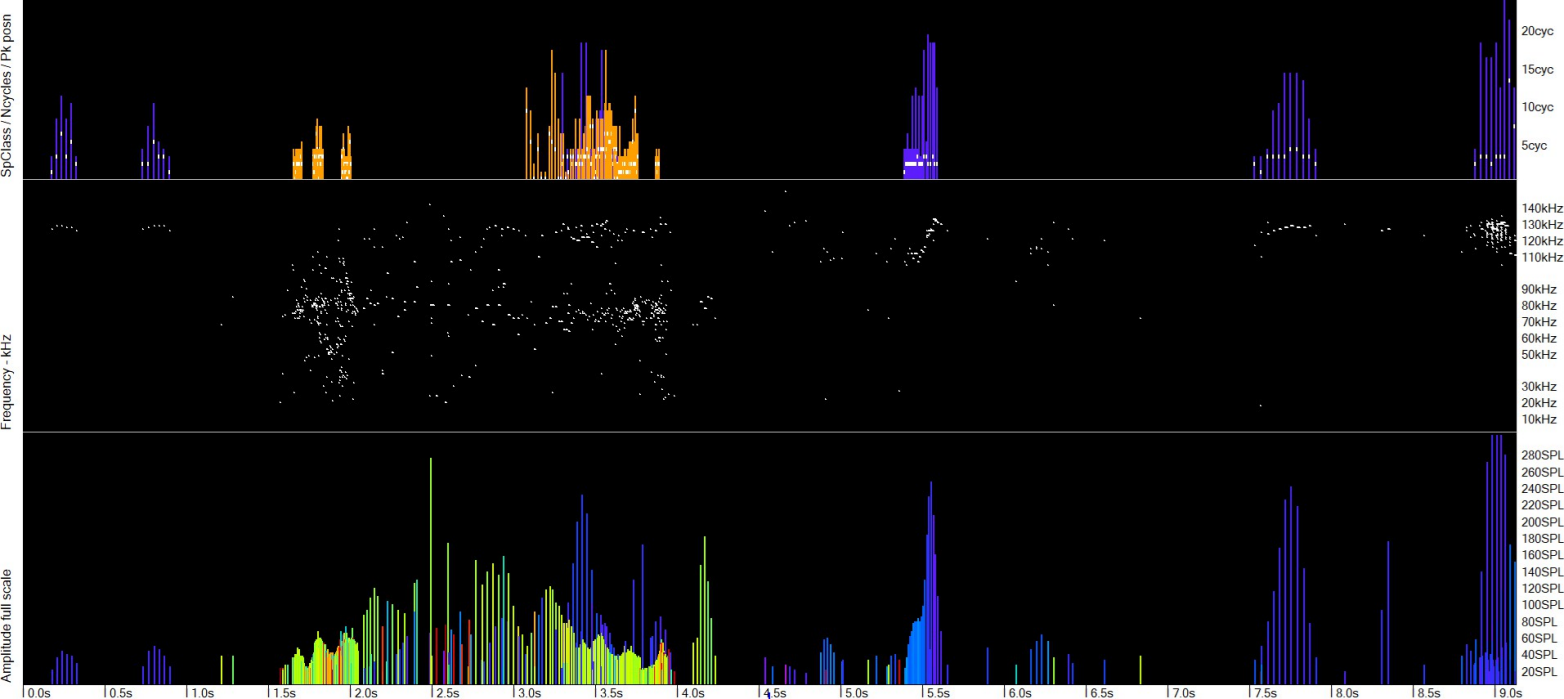
Number of cycles and click frequencies in the raw data. The upper panel shows the number of cycles in each click and the species identified: orange = dolphin, violet = porpoise. The middle panel shows the click frequencies in the raw data, and the amplitudes are shown in the lower panel, as vertical lines colour-coded by frequency. Here porpoise and dolphin echo-location overlap and the frequency display is consistent with the classification shown in the top panel.

**Fig 8:**
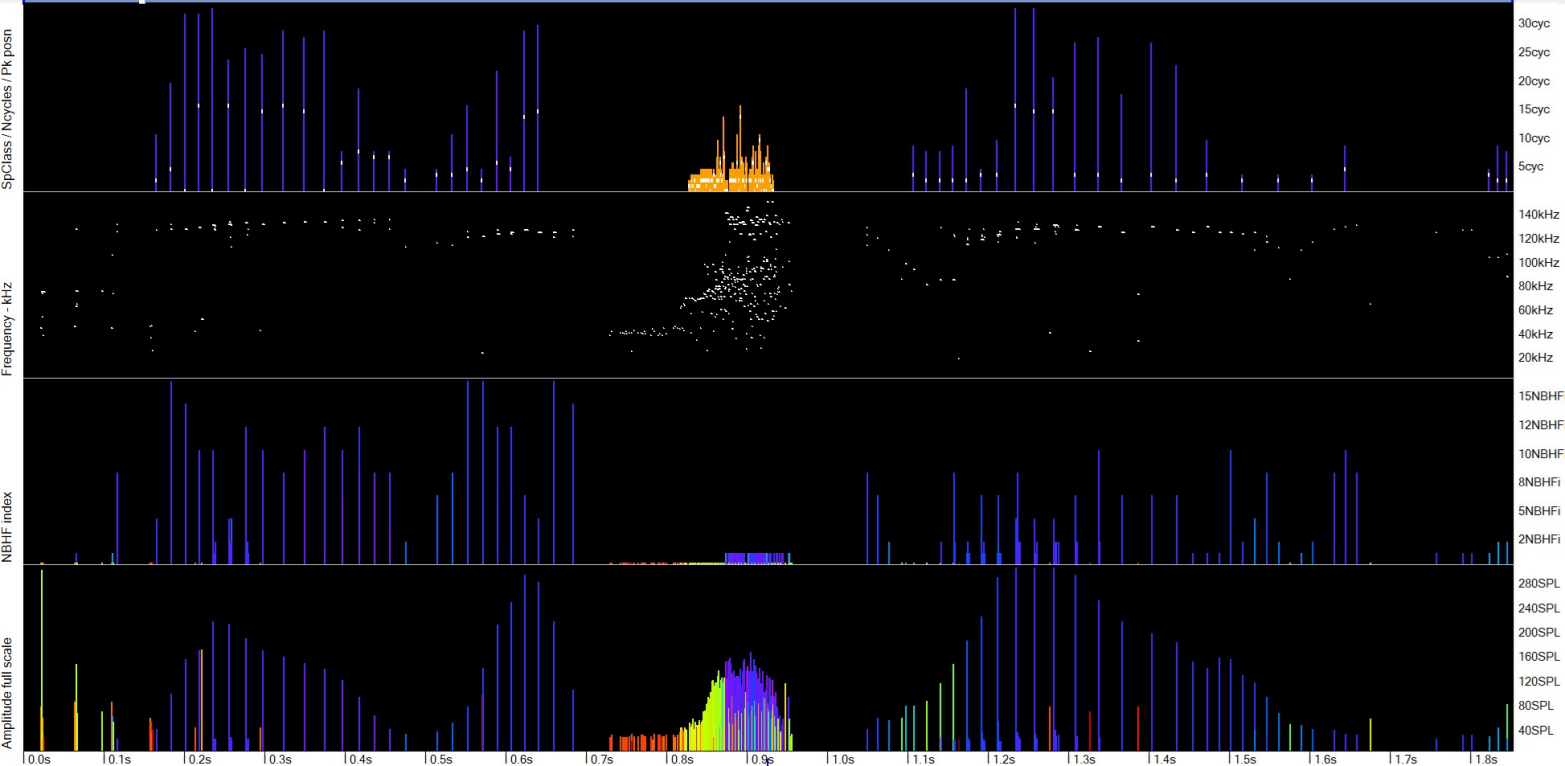
The utility of the NBHF index in determining species where they occur together. The lower three panels are all raw data and show a click train in the centre that has clicks at porpoise frequencies with typical porpoise click trains just before and after. The NBHF index for this central click train is less than 3 (mostly 1). The classification of this as a dolphin click train, shown in the upper panel (violet = porpoise; orange = dolphin) is thus likely correct.

**Fig 9:**
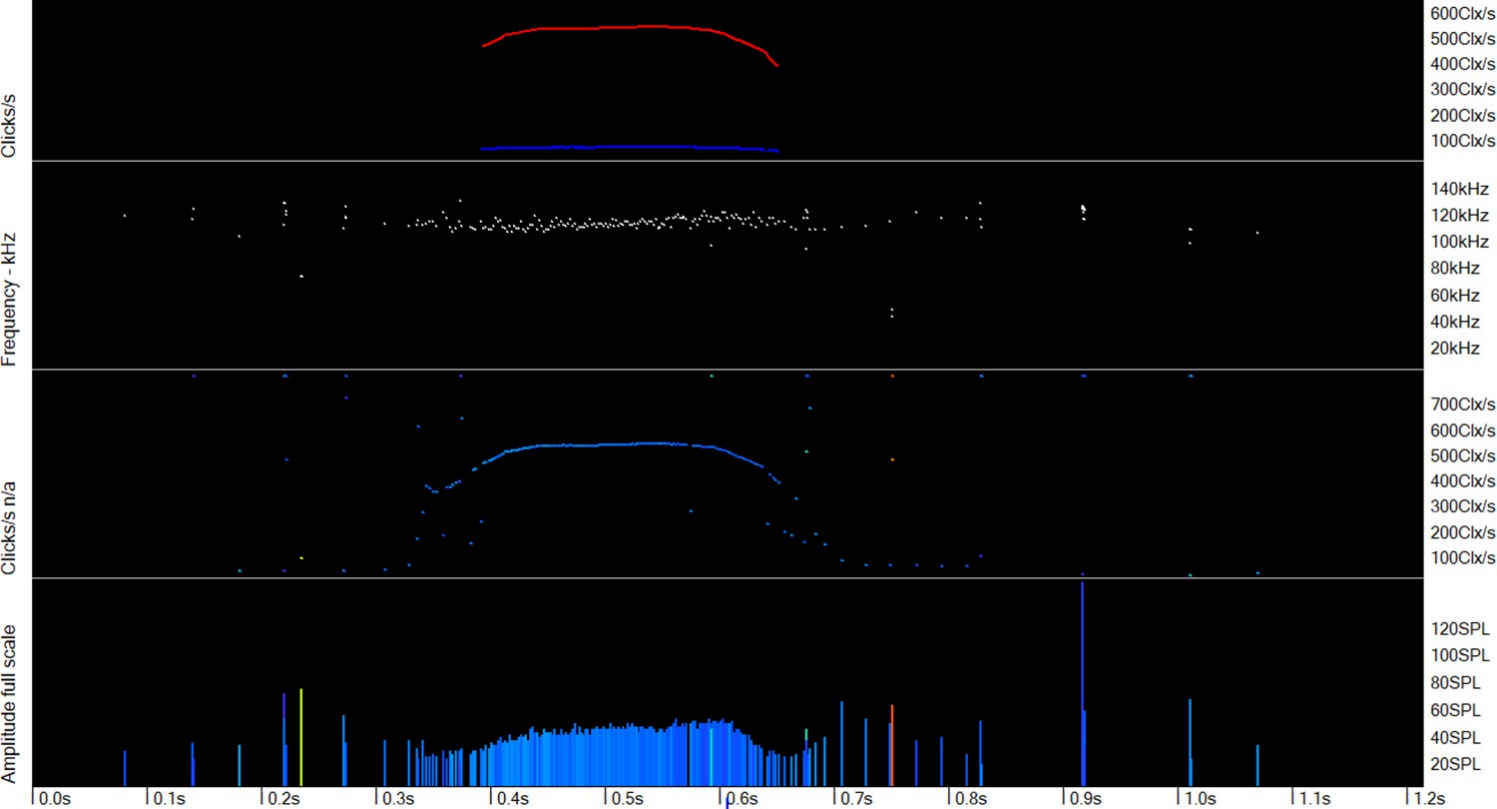
A weak unknown train source (WUTS) that was misclassified as a dolphin. The lower three panels show the raw data. The train is preceded and followed by very low click rates in a nearly symmetrical pattern that is very unlike a cetacean. The KERNO-F classifier did not identify these tails as belonging to the train because they show changes in successive inter-click-intervals that are outside the range of click-rate change that the classifier will accept.

**Fig 10:**
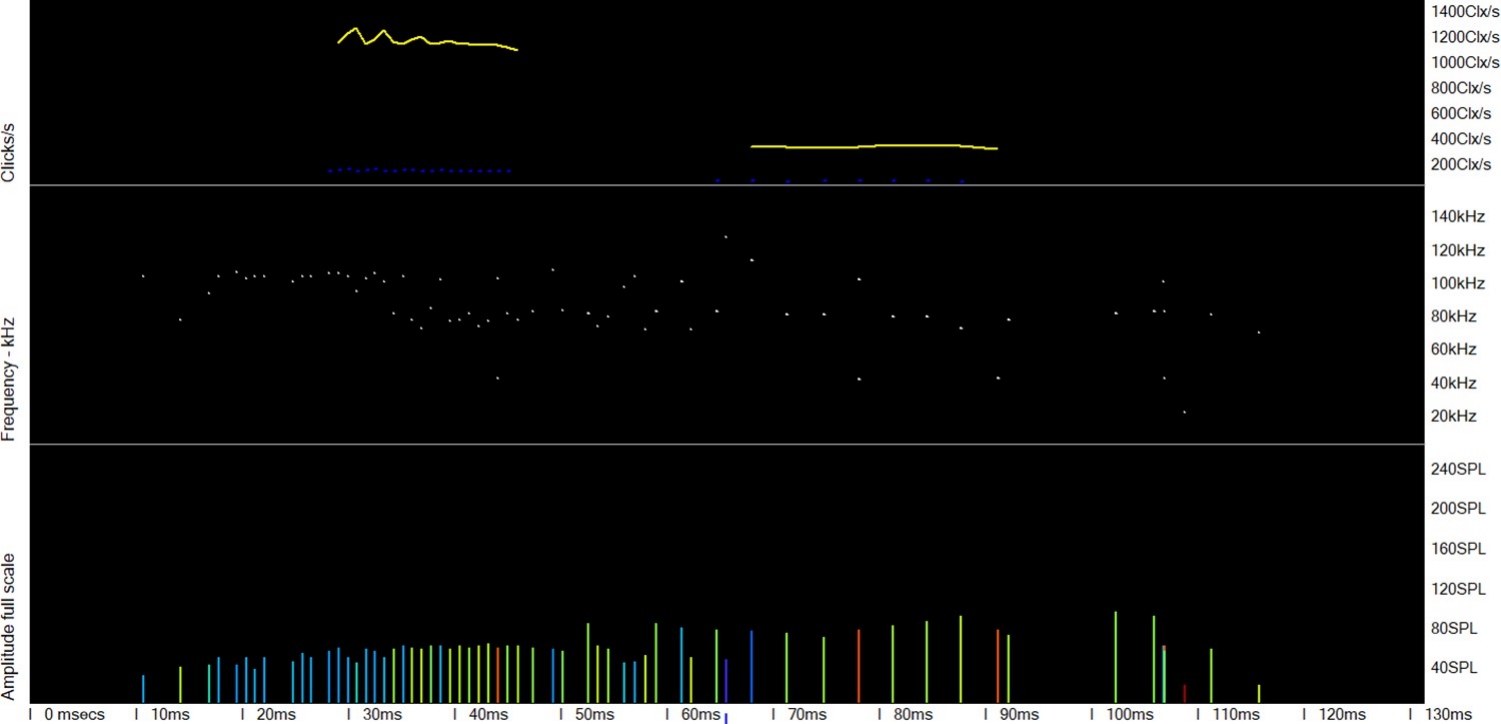
A weak unknown train source that was misclassified as a dolphin. The lower two panels show the raw data. In the top panel, the train shows very high click rates and then a sharp step down in click rate. This, combined with many large changes in frequency (mid panel) between successive clicks, is unlike a cetacean.

**Fig 11:**
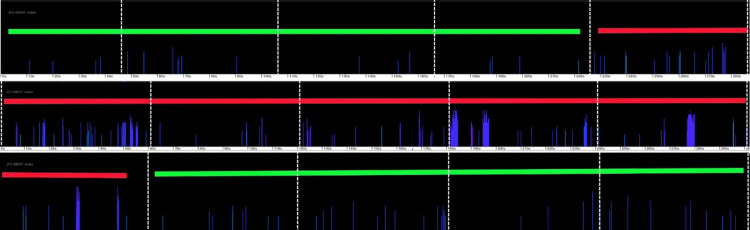
Time sequence of a porpoise encounter shown in a raw data file, starting top left. The horizontal axis shows all clicks in 15 minutes of data in three panels of 5 minutes duration, marked by vertical dashed lines at each minute. The vertical axis is the narrow-band high frequency index (NBHF index–this represents how closely each click matches a typical NBHF clicks as produced by a porpoise). Each coloured vertical line is a click but many overlay others at this time resolution. The first section, marked in green, shows background noise. During the next 7minute section, marked in red, a series of increasingly distinct groups of clicks with higher values of NBHF index are logged and then the pattern reverts rapidly to the background noise pattern, again marked in green. This evidence of a typical encounter adds support to the classification of a train within that likely encounter as being a porpoise click train.

The evaluation process was carried out by viewing the click trains in the FP3 file alongside the raw data in the FP1 file and determining the validity of the classification assigned by the KERNO-F classifier by assessing each of the following features of the data and making a judgement on the overall picture:

1. For the DPM to be assessed as correct, other plausible click trains or train fragments from the target species category should be present within the three minutes either side of the focal train.
2. Clusters of weaker “replicates” arising from multi-path propagation (see Fig 2) commonly follow cetacean clicks and boat sonars. The frequency profile of these clusters is a useful feature: In porpoise click trains these replicates are generally within the porpoise frequency range, here considered as 100-150kHz. In dolphin click trains the similar clusters of frequencies, differing from the direct path (the first click) is commonly seen. This occurs where the off-axis parts of the dolphin’s sound beam reach the logger by reflection from the sea surface. Patterns such as those in Fig 3 are strongly indicative of a dolphin as the source and are a positive feature. Boat sonars often show large clusters of very long clicks.
3. Successive multi-path replicate click clusters in cetacean or boat sonar trains show a degree of coherence in patterns of delay and amplitude as well as frequency. So widely differing patterns may indicate that the train consists of clicks from different sources (see Fig 4).
4. Inter Click Intervals (ICIs) seen in the raw FP1 file, or in all the click train categories in the FP3 file, should not be stable over one or more minutes. A line of nearly constant ICIs is indicative of a sonar misclassified as animal click train. Fig 5 demonstrates the identification by the classifier of a sonar at 120 kHz, which is also a typical harbour porpoise frequency [39]. When the click rate in the FP3 file had been stable for more than a minute, which is unusual for living organisms, the classifier gave a correct classification of the sound as sonar. In this case the algorithm treated the sound, incorrectly, as a harbour porpoise because the boat sonar was just coming into the detection area of the F-POD or fading away.
5. The profiles of click amplitudes from porpoises typically have smoothly rounded envelopes of discontinuous bursts associated with the cetacean sonar beam sweeping across the hydrophone (see Fig 6). This also applies to weaker dolphin click trains. Such envelopes support cetaceans as being the source of the train. However, louder dolphin trains often saturate the limited dynamic range of the F-POD and then this feature is not expected.
6. The frequency display of the FP1 file, as shown in Fig 7, shows the frequencies of all the clicks. Where a distinct pattern of two different groups of frequencies is seen this supports a classification of two species being present with a short period or even overlapping.
7. The NBHF index is a composite index based on the wavelengths, number of cycles, wavenumber of the loudest cycle in the click, number of amplitude trend reversals in the click envelope, bandwidth, and range of wave periods within the click (see details in S1 File). For porpoise click trains, the NBHF index should be greater than three in most trains within a minute. For dolphin click trains, the NBHF index should be mostly less than three. Fig 8 shows the utility of the NBHF index in determining species where they occur together.
8. In cetacean click trains the position of the loudest cycle in a click usually shows some coherence through the sequence of clicks, while in sonars and click trains arising by coincidence from multiple sources it typically shows very little coherence (see Fig 5).
9. Click trains logged are typically fragments of longer trains captured as the cetacean sound beam sweeps across the hydrophone. If the start or end of a click train can be identified by simultaneous trends in amplitude and click rate or click kHz, it is unlikely to be from a cetacean. Recognisable exceptions are social click bursts [34] in which the amplitude profile and click rate can show strong correlations e.g. the click rate rises sharply with the amplitude at the start and they decline together at the end. Weak Unknown Train Sources, which are currently unidentified (WUTS) also produce examples of such patterns and may also show unusual click rate profiles such as long slow descents from very high click rates. These atypical patterns are a useful part of visual validation. Figs 9 and 10 show WUTS that were misclassified as a dolphin trains.
10. Cetaceans are rarely stationary and usually generate “encounters” that can be identified in the acoustic data. A typical cetacean encounter, as in Fig 11, begins with weaker clicks and click train fragments appearing, becoming stronger and clearer over a few minutes as the animal approaches and then becoming weaker, less distinct, and disappearing generally much more rapidly than they appeared. In contrast, boat sonars or other man-made sources typically show a symmetrical time profile, and weak unknown train sources are usually sporadic events without any such pattern over time.

## Results

Data were collected from 21 stations between 26 September 2020 and 31 October 2022. During this period, 87 files comprising 6,418 logged days and 9,049,060,966 clicks in total were generated and are listed in S1 File.

The KERNO-F output is shown in Table 1. The mean number of click trains in a DPM (detection positive minute) was 10.4 porpoise trains and 10.7 dolphin trains, composed of 225 and 245 clicks, respectively.

**Table 1:** Data set: All detections.

*Data*:	Porpoise detections	Dolphin detections	Sonar detections	Unclassified
Number of clicks	297,122,376	91,955,342	5,802,174	45,721,371
Number of trains	13,710,490	4,010,704	159,540	2,042,785
Detection Positive Minutes	1,323,298	374,974	50,364	444,806

**Fig 12:**
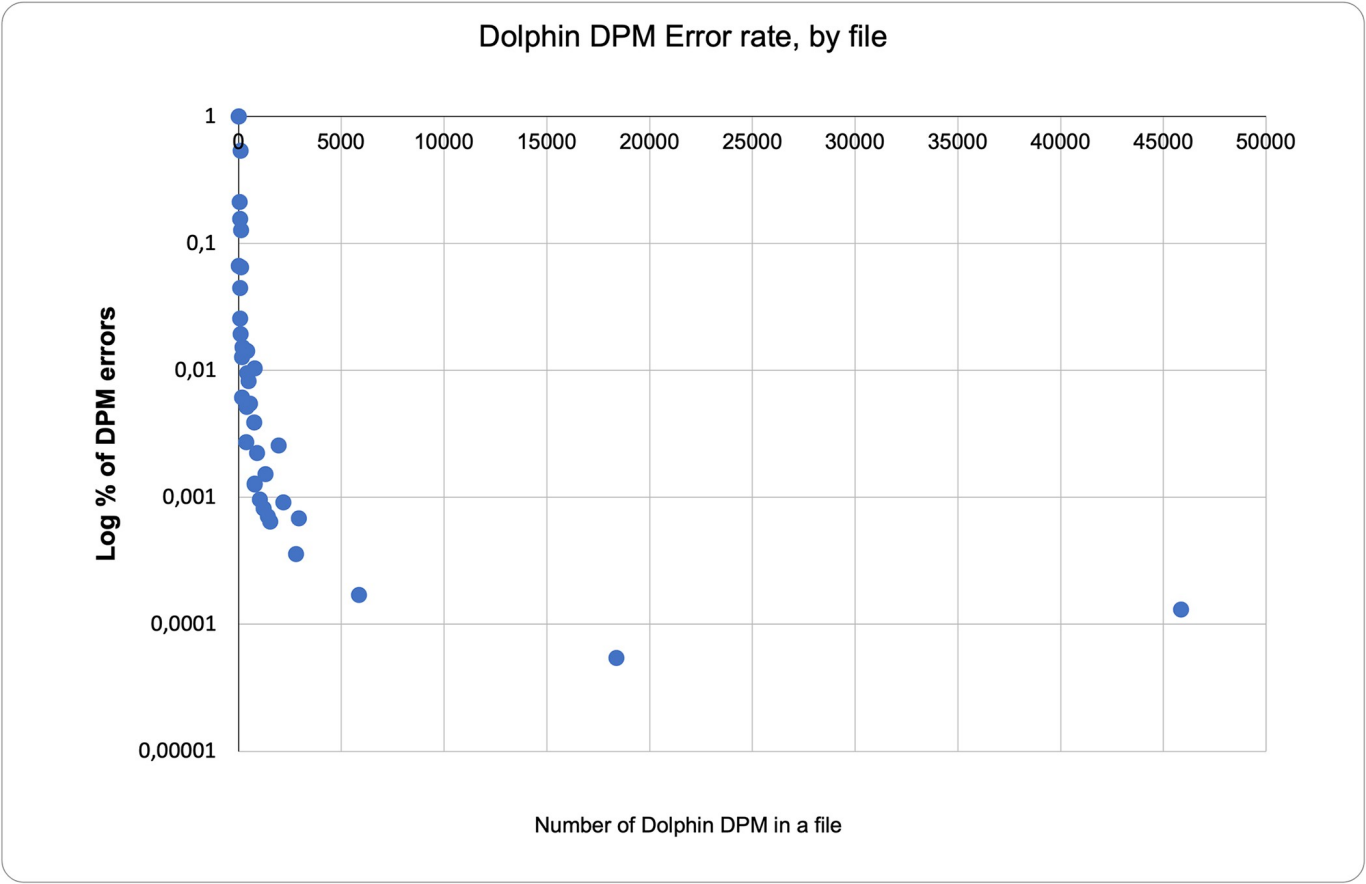
Distribution of Dolphin error rates across files.

Approximately 168,000 click trains were inspected. Table 2 shows the prevalence of false positive errors across the whole project and in each file based on the evaluation described. The higher rate of errors per DPM found in the examination of individual files is expected because of variation in the number of DPMs per file. For example, for two files with the same number of false positive DPMs (for instance 100) but a different number of true DPMs (for instance 10 and 1,000), the file with fewer true DPMs will have a higher false positive error rate per DPM (100/110 = 91%) than the file with more true DPMs (100/1,100 = 9%). In the extreme case, which did not occur in this study, a sampled file that has no true DPMs must, if any false DPMs are present, give 100% false error rate per DPM. Fig 12 shows the non-linear distribution of false positive error rates for dolphin detections as a percentage of total DPMs per file.

**Table 2:** False positive errors identified by the evaluation process. DPM = Detection Positive Minute.

*Data*:	Porpoise detections	Dolphin detections	Sonar detections	Unclassified
*Error rates in the whole project*:				
Number of DPM evaluated	2000	2000	0	2000
Total number of errors found	0.06*	19.4*		
% DPM with a false positive click train	<0.01%	0.97%		
*Error rates in individual files*:				
Number of DPM evaluated	8,548	8,257	0	0
Total number of errors found	37	169		
% DPM with a false positive click train	0.43%	2.05%		
Range of error rates per file	0–29%	0–48%		

* these factional values arise from the downscaling of the number of errors found in some files as set out in the description of the two sampling methods.

Table 3 shows the sources of all the errors found during the process of DPM evaluation in the full set of 87 data files. These sources of error do not include any report of a ‘chance train’ arising from noise but misclassified as a cetacean. No examples of this were found even though approximately 168,000 click trains had been inspected. In three porpoise DPM, after consideration of all the features discussed, the minute was rejected because the true species remained in doubt although the classification as porpoise was still considered to be the most likely source by the assessor.

**Table 3:** Sources of errors found in sampling cetacean detection positive minutes.

*Species group classified*	*Source of error*				
	Porpoise	Dolphin	Sonar	WUTS	Files with errors
Porpoise	-	0	34	0	2/87
Dolphins	143	-	17	9	38/87

WUTS = weak unknown train sources.

The error rates generated by specific sources were counted in a set of files to determine the rate at which each possible source (porpoise, dolphin or sonar) leads to a false classification of one of the other species types. These files had low counts of the ‘other species’ making it feasible to check them all for errors. Table 4 shows the results. The number of instances found in this data set of sonars classified as dolphins, or of dolphins classified as porpoises, was too low to give a useful specific error generation rate.

**Table 4:** Source specific false positive error rates per DPM in selected files.

Source	No. of files	Total DPMs	Misclassification to:	DPMs with false positive errors	% DPM with errors
Porpoise	9	45,232	Dolphin	63	0.14%
Sonar	9	391	Dolphin	5	1.28%
Dolphin	4	7,474	Porpoise	0	0.0%
Sonar	2	19,843	Porpoise	34	0.17%

**Fig 13:**
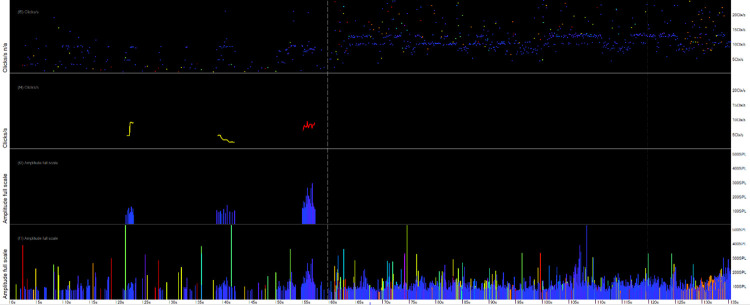
Example of sonar mis-classified as a porpoise.

Table 5 shows the results from the two files that had the only detected errors from sonars misclassified as Porpoises. They also had the lowest number of Porpoise DPM, all of which were validated because there were fewer than 100. The same two files also had a rate of Sonar DPM/day that was more than twice that of the next highest in the whole data set. These two factors contributed to the high number of false positive DPM and allowed an estimation of the rate of misclassification of sonars as Porpoise for each file. A typical example of this kind of error is shown in Fig 13.

**Table 5:** Error generation rate for sonars misclassified as porpoises.

*file*	Porpoise DPM evaluated	DPM from sonar	DPM Unclassified	Sonar DPM	% SONAR DPM misclassified as Porpoise
T Istanbul 02 2021 07 14 FPOD_6314 file1 PART 49d 8h 17m	74	27	(2)	15828	0.17%
T Istanbul 02 2021 07 14 FPOD_6314 file2 PART 23d 14h 55m.FP1	76	7	(1)	3601	0.19%

Lowest panel demonstrates amplitudes from raw logged data (.FP1 file). Second panel up–amplitudes of click trains classified by KERNO-F classifier (.FP3 file). Third panel–click rate of classified click trains in clicks per second (.FP3 file). Top panel–click rate of all clicks from raw logged data (.FP1 file) showing evidence of a persistent constant click rate.

### Evaluation of validation process

The two files in Table 5 were independently evaluated by a second assessor. These files were selected because they had sufficiently few DPM with Porpoise detections (74 and 76) combined with a very large number of sonar DPM (15,828 and 3,601 respectively, while the total number of sonar DPMs in all files was 50,364). Both assessors found the same number of DPMs in each file (29 and 8) that were considered clearly false or should have been ‘unclassified’.

### False negatives

To assess the incidence of cetacean or boat sonar click trains placed in the ‘unclassified species’ class, 2000 DPM, distributed evenly across the set of files, were inspected and each unclassified train was reassigned to a species class where possible. Each minute had 1 or more trains of ‘unclassified species’ and was consequently a DPM for an ‘unclassified’ train. In such a minute multiple unclassified trains may be present and may be reassigned to more than one of the species groups. The results are shown in Table 6 with the correlation, across the 87 files, between the number of DPM in the file for each species group, as assigned by the KERNO-F classifier, and the number of minutes in which an unclassified train was reassigned to each species group by the human analyst. In the whole set of 87 files the number of clicks in unclassified trains of high or moderate quality (45,407,756) was 10.4% of all clicks in all classes.

**Table 6:** False negatives: Analyst reassignment of “unclassified species” trains in 2000 detection positive minutes.

	Porpoise	Dolphin	Sonar	Unclassified
Species	1,993	721	407	61
Pearson Correlation between N of species DPM in file and N of of reassigned minutes	+0.90	+0.93	+0.96	+0.71

## Discussion

In any large quantitative click monitoring project the questions that the project seeks to answer, and the precision required, determine what levels of error can be accepted in the automated output. We have not addressed those statistical issues here but chose 2000 samples spread across the detections of a species group. The effect of this arbitrary sample size is that if errors were randomly distributed across DPM this would, by simple probabilities give approximately a 95% chance of capturing at least one error in the sample of 2000 DPM if the error rate was above 0.15%. The error rates found here are of that order.

Errors will not be randomly distributed across monitoring sites because very significant features, such as the prevalence of boat sonars or sediment transport noise, are likely to vary between sites and boat sonars can vary greatly over time. The assessment of individual files illuminates this issue and, in this study, showed that one site with very high boat sonar prevalence also had very low porpoise detection rates. This site-based assessment could be used, early in a project, to filter the sites to be included in the assessment of different research questions.

Beyond these project-specific issues it would be useful to be able to predict the risk, in any project, of errors at specific sites or the whole project, by getting a source-specific error generation rate for sources that can themselves be quantified in the acoustic data. We have not found any published assessments of this for PODs or other acoustic methods. The sources we have tried to assess here for F-PODs are cetacean groups, and boat sonars, but sediment transport could be included as minutes are marked by the KERNO-F classifier in F-POD data if they have some source of continuous noise. Such sources would produce ’chance’ trains, and none were found in this validation work, so we have not taken this further. The assessment of the reliability of these source-specific error rates requires study of data from diverse sites that mostly do not have high levels of true positives as these hide the false positives as Fig 12 shows. However, the similarity of the two rates shown in Table 5 is at least encouraging, and we submit that this approach to reporting the performance of classifiers is widely applicable and more useful than global error rates expressed as a fraction of true positives plus false negatives.

The data set of the BlackCeTrends project has a relatively big number of detections of both dolphins and porpoises. Such high levels of true positives make it difficult to find false positives because they are among much larger numbers of true positives. By contrast, if one species group was absent then all detections of it would be false, and the rate at which they were generated by different sources as boat sonars, could be easily determined.

Fig 12 shows the relationship between error rate and dolphin DPMs. A significant implication of this is that when detection rates are low the validation of each one becomes more important, but also becomes quicker because there are fewer.

Two different measures of false positive error rates have a particular value:

1. A reliable estimate of the *overall false positive error rate* of the operational statistic for a specific study is needed to decide whether the data from the classifier can be used without editing in that study. The very low overall error rates found here in the BlackCeTrends data support the use of this data for year-on-year trends or other questions without editing.
2. *Source-specific rates of generating false positives* in the operational statistic are valuable as they potentially enable users of this classifier to identify times where error rates at particular locations may be unacceptable because of the prevalence of the source of errors, and to compare the performance of different classifiers or settings of classifiers, or to model the generation of errors more directly than when errors are expressed as a fraction of true positives that are essentially independent of them.

F-POD data shows the prevalence of three of the sources of species errors: porpoises, dolphins and boat sonars, so rates of generation of errors by each can be assessed and are shown in Tables 4 and 5.

The rate of misclassification of sonars depends on their frequency (kHz) so the detected sonars in a file could be filtered by their frequency to improve the prediction of the rate at which false positive NBHF clicks might happen.

Establishing any benefit of quantitative models of likely errors will require similar analysis of data sets from widely different acoustic contexts, preferably including some with low true-positive rates to make the determination of false positive rates easier.

The prediction of an error generation rate from ambient noise, which can by chance generate spurious trains, is not possible because the characteristics of such noise are diverse and will strongly affect the generation of error rate. In this study no false positive errors arising from non-train sources were identified. However, a known problematic example is the transport of sand which, depending on the dominant particle size, generates bursts or periods of sound at NBHF or lower frequencies [39]. In a study comparing instruments (C-POD and Soundtrap + PAMGUARD) Sarnocinska *et al* [51] found very large discrepancies between the output of a click-by-click classifier, PAMGUARD, which uses a click-spectrum based classifier of broad-band data and sometimes reported very high levels of ‘porpoise detections’ while co-deployed C-PODs analysed by the KERNO classifier reported no click trains. The evidence from this study suggests that the explanation of this significant discrepancy is likely to be misidentification of sediment transport [50], which may be linked to using a lower detection threshold giving a potentially greater detection range.

For the BlackCeTrends project this analysis gives insight into what factors may cause the error rate to vary over time and this can give indications of which sites are less suitable for analysing trends, with high levels of boat sonars flagging up a risk of changes in animal detection rates that may arise as the frequencies of sonars or their prevalence change over years. The data identified such a site that combined the highest incidence of sonars with the lowest incidence of NBHF detections. It gave all the errors in NBHF data found in the survey of the individual files, so there is a case for using such validation results to reassess some sites that are too acoustically difficult.

Weak unknown train sources are not identified as a separate category by the KERNO-F classifier and the rate at which errors arise from them can not be readily estimated. This is a significant deficiency that is due in part from the lack of examples of WUTS during the development of the classifier and to a lack of knowledge of these sources. In this project they were rare but, in the absence of any substantial account of these sources, vigilance in the form of systematic data validation, is needed.

False negatives represent a very different challenge from false positives. They fall into two categories:

1. Unidentified trains where the clicks are logged but do not reach the threshold in the KERNO-F classifier for being placed in the cetacean or sonar species classes and in the High or Moderate train quality classes.
  The strong correlation in files (Table 6) between the reassigned species group of the unclassified trains and the detection rate by the KERNO-F classifier of that species group shows that the ‘unclassified’ species group essentially represents a loss of sensitivity, i.e. a reduction in the detection performance, rather than being some other source that might be categorised as ‘noise’. The common occurrence of multiple species in these minutes points to a likely mechanism within the classifier i.e. click trains of multiple species groups, close together in time, biases the classifier against attributing a species to any of them.
  Those that are identified as low-quality trains, or not as trains at all, are difficult to quantify because they include trains with an increasing prevalence of missing clicks. These can increase until the train is only represented by 1 or 2 clicks that are indistinguishable from many clicks resembling cetacean clicks that are logged from non-cetacean sources. As a result, this assessment is very subjective and has not been attempted here.
2. Click trains that were not logged because the animal was too distant or facing away from the logger. Here an ’effective detection radius’ (EDR) for a suitable time window could express the distance detection function by defining a radius with as many detections made outside as were missed inside. That is a field task requiring tracking of the position of the animals in the vicinity of the logger.

Obtaining a detection function has been attempted for C-PODs for Baltic harbour porpoises in SAMBAH project [15], for T-POD and Hector’s Dolphin [52] and Bottlenose Dolphins [16, 53]. The directionality of the cetacean click beam means that it is mostly not detectable by any logger even when the animal is well within detection range. Over time the animal is more likely to be detected as it sweeps its sonar beam around its environment. In line transect studies a time factor is always introduced by the speed of the vessel, while in static monitoring a time window for detections must be specified and will be relevant to the application of the function to the data.

Studies such as the Vaquita population trend monitoring proceeded without a detection function on the basis that the determinants (as above) were likely to be similar in the years studied and it was reasonable to assume the detection function would stay similar and trends could be assessed [17].

Sonars may also affect the actual local distribution of cetaceans and could be viewed as a source of false negatives. This effect has been shown for fishery pingers [25].

## Conclusions

In two years of data from the BlackCeTrends project the error-rate for harbour porpoise detection positive minutes was <0.01% and the error-rate for dolphins was 0.97%. With such error-rates we can use the data from the BlackCeTrends project, without editing, for investigations in diel and seasonal patterns of cetaceans in the Black Sea and potentially for long term trends without editing.

The errors found were highly structured, and source-specific error generation rates were found. When these have been more widely assessed, they may be useful for other projects involving F-PODs.

Each data file needs manual validation in case other sources of errors appear and to identify files that may come from unsuitable sites. The available software tools make this a feasible task.

## Supporting information

S1 File (DOCX)

S2 File (XLSX)
