# Supplementary material for: Validation of the F-POD—A fully automated cetacean monitoring system
Source: PLoS One. 2023 Nov 17;18(11):e0293402. doi: 10.1371/journal.pone.0293402 (PMC10656029; doi:10.1371/journal.pone.0293402)
Supplement: S1 File — (DOCX) [file pone.0293402.s001.docx]

F-POD technical information

# Data capture

The signal processing is structured to give low data volumes and match the requirement of the post-processing which is based on the detection of trains of clicks. The functional modules in the F-POD hardware and post-processing are shown in Fig A.

*
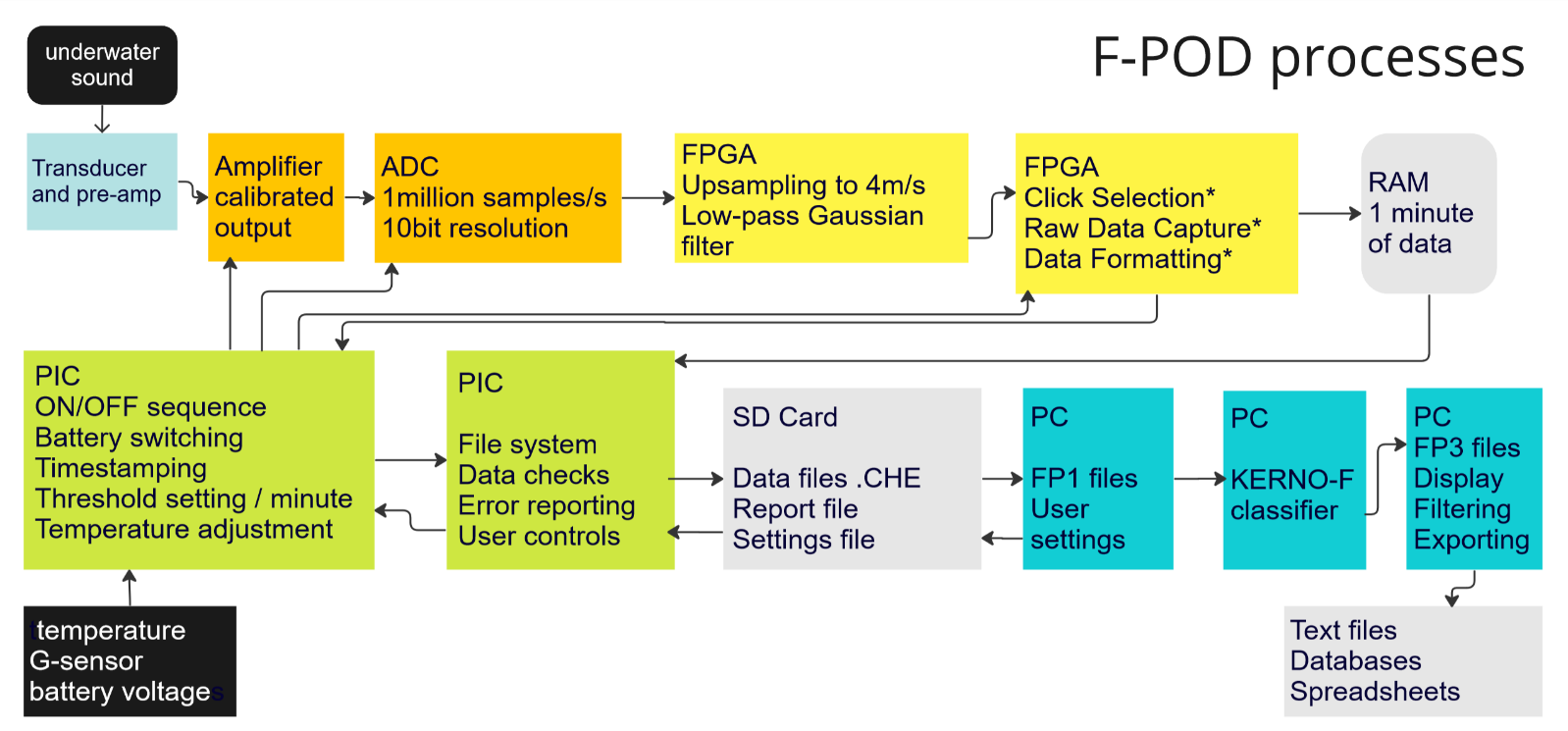
*

Fig A. In this sequence all items shown up to the SD card are within the POD itself and the elements in blue are post-processing on a PC. Abbreviations used are: ADC: Analogue to Digital Converter, FPGA: Field programmable gate array micro-controller, PIC: Peripheral Interface Controller, RAM: random access memory.

*Transducer and pre-amplifier:* The transducer (hydrophone) is a single piezo-ceramic cylinder. The pre-amplifier, and subsequent processing, apply a high-pass filter to reduce sounds below 20kHz.

*Amplifier calibrated output / Gain*: This gain value is set at calibration and is varied during operation by a small amount in response to temperature to compensate for the change in acoustic impedance of the transducer housing.

*ADC – analogue to digital converter*: the sampling rate is fixed at 1m/s.

*FPGA:* The digital data stream is upsampled in real time by zero-stuffing to 4 million/s followed by a Gaussian low-pass filter to give 250ns resolution in the timing of individual inter-peak-intervals (IPIs).

The effect of up-sampling, low-pass filtering, and peak designation is illustrated in Fig B. Clipping is commonly encountered in cetacean clicks in this system but has little effect because the low pass filtering restores a peak to the flat top. Information on clipped amplitudes is retained in the 8bit amplitude value and allow some retrospective estimation of the true peak height, but this is a noisy value, particularly for high frequencies.


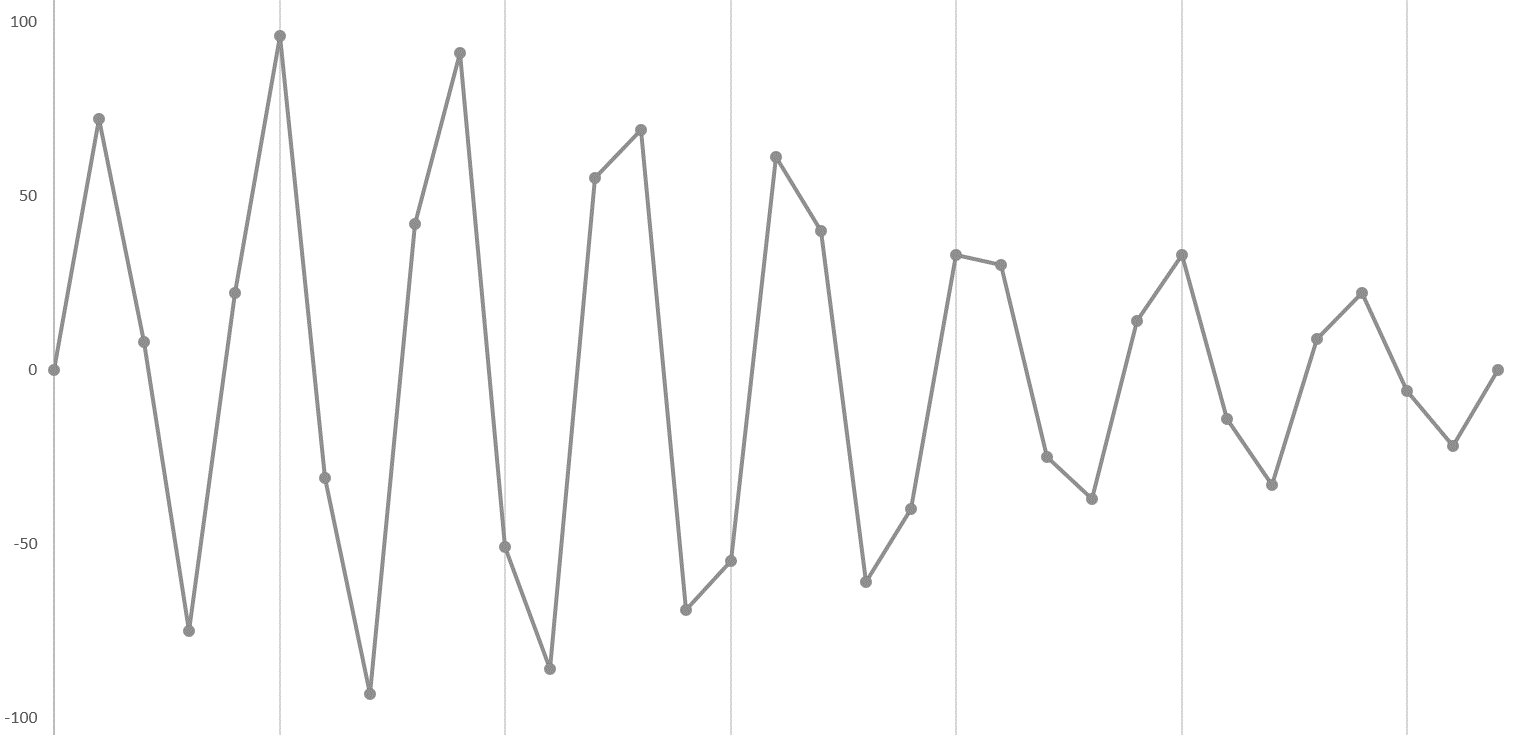
Wave-peak identification: Peaks are confirmed if the amplitude falls below half the peak.


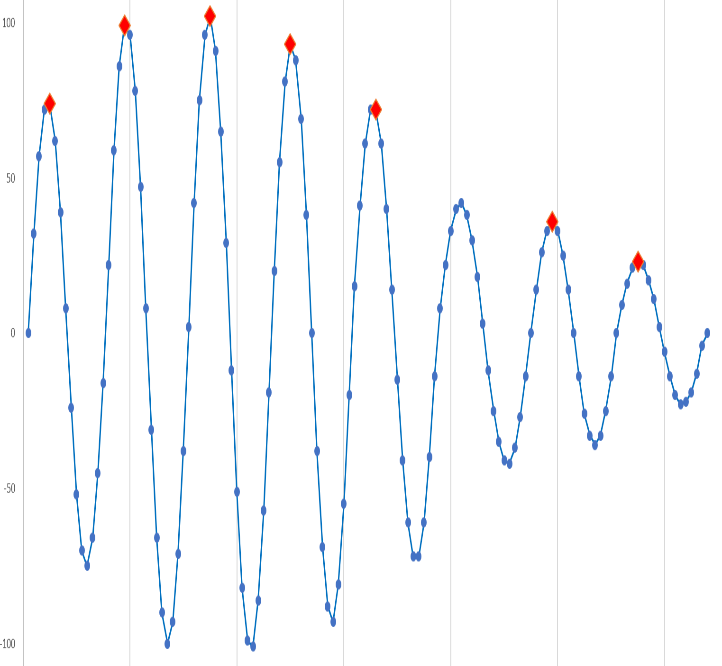


Fig B Left: a click sampled at 1million samples per second, and Right: after up-sampling to 4m/s, and low pass filtering with the utilised peaks marked. This process has corrected the wave number of the loudest cycle in the click, and the number of reversals of the amplitude trend.

*FPGA:* This Field programmable gate array micro-controller carries out parallel processing to enable it, a low-power chip, to complete all the computations on a click between one wave peak and the next in the incoming data stream. Successive inter-peak-intervals are calculated. Frequencies from 17kHz to 220kHz are admitted to the click selection process. These limits can be varied by the user. The ratio of change from the prior IPI is calculated.

Click extraction: a click continues while successive IPIs are less than approximately 50% of preceding IPIs. This is a purely tonal criterion.

Click selection: to be stored an extracted click must exceed an amplitude threshold. This threshold is the same for all clicks of 5 more cycles and increments successively for clicks of 4,3,2 and 1 cycle. The steps are user-defined.

Features of clicks are tracked including the position of the loudest cycle in the click, the range of IPIs in the click, the number of reversals in the trend of cycle amplitudes, the last IPI value, the IPI before the clicks start, the amplitude of three waves around the highest, and the time of the click. Referencing IPIs and amplitudes to the loudest cycle preserves significant power in time-domain matching of successive clicks in a train.

Raw Data Capture: some clicks, typically less than 1%, are recorded in greater detail. The time and amplitude of up to 21 cycles of the click are logged. It is then possible in post-processing to fit sine waves to these peaks and generate a WAVE file snippet. This functionality is not used by the KERNO-F classifier or in any of the analysis of the data presented in this paper.

Sonar detection: active boat sonars can be identified in real time using the extreme uniformity of their IPIs. If found an exclusion band is set around that frequency. Two independent sonar detectors can operate. Both are reset each minute.

Data formatting: Each click record is in 16 bytes containing the following feature data:

0 time within minute in 5 microsecond units, MSB

1 time in 5 microsecond units

2 time in 5 microsecond units, LSB

3 N of cycles in the click - up to 255cycles

4 Wavenumber of loudest cycle; range of IPIs in click

5 IPI  of Pk-1

6 IPI of  Pk  this is the loudest cycle in the click

7 IPI of  Pk+1

8 IPI of  Pk+2

9 Amplitude of  P-1

10 Amplitude of  Pmax,  the loudest cycle in the click

11 Amplitude of  P+1

12 IPI before click start

13 N of Amplitude Reversals in click envelope; Duration of click (MSB)

14 Duration of click (LSB)

15 IPI of last cycle, compressed; flag if boat sonar found

This output differs radically from standard WAVE files, which contain a sequence of amplitudes measured at evenly spaced points in time as in Fig B.  Consequently, the Discrete Fourier Transform and similar processes cannot be applied to POD data, and instead time domain methods are used. Various features are derived from the parameters listed above.

*PIC*: this micro-controller carries out various essential ‘housekeeping’ functions and sets the threshold for each minute against user-defined settings:

Threshold Setting: if the number of clicks logged in a series of minutes exceeds a set value the criteria for storing a click are adjusted upwards to the next set of criteria in a series of steps. Initially the amplitude thresholds for logging clicks that have less than 5 cycles is raised. This continues with subsequent steps with the threshold for clicks of 5 cycles staying low except in very severe noise. The rationale is to exclude those clicks that have the lowest chance of being useful in train detection or may start to impair that process. A similar process of stepping down the threshold settings occurs if a series of minutes have fewer than some user-defined number of clicks.

This threshold adjustment largely avoids loss of logging time due to limits on the number of clicks permitted in a minute (‘maxing out’) without changing the gain or amplitude scaling.

# Post-processing: Train detection

*Creation of the FP1 file:* The FP1 file is essentially a copy of the .CHE file on the SD card plus a larger header holding location and other data from the user, and with some data integrity checking that can mark defective records (now very rare). It is subject to the train detection process that generates the .FP3 file, containing only those clicks that are found to be in trains.

The term train is used here in the sense of a series of similar elements with some similarity between successive inter-click intervals. The elements in this case are clicks and their features and the inter-click intervals may change progressively and substantially through the train.  Such trains can come from odontocetes, from some unknown biological sources, and from boat sonars. Logged dolphin clicks can show strong changes in characteristics as the dolphin’s sound beam sweeps across the hydrophone because the sound in the centre of the beam (on-axis) can have a substantially different frequency content from the sound further away from the beam axis.

*The KERNO-F classifier*

This classifier carries out the train detection on a Windows platform.  It is an algorithm that systematically seeks click ‘trains’ in the series of clicks logged in each minute. It is not based on machine learning methods or neural networks, but uses the earlier feature engineering approach in a layered and highly recursive structure combined with multiple hypothesis testing and several processes that carry out heuristic ‘load-shedding’ functions. That is essential as the computational load of a ‘brute-force’ approach to even 10,000 clicks (commonly logged in less than 1 minute) would have to find and evaluate more possible solutions than the number of seconds since the origin of the universe. To handle the computational load the algorithm uses only integers and scales these where required to obtain appropriate precision. It is written in a version of Pascal (Delphi).

The first of the load-shedding functions is an initial process that seeks to identify clicks split by acoustic refraction along the pathway from source to receiver. This is done by passing a very short time window through the data. Later in the process a longer time window of variable length seeks to identify and characterise the multipath clusters that commonly accompany clicks from loud distant sources, as shown in Fig E. These are highly significant as similarity of successive clusters is a powerful indicator of a common source, which helps to exclude trains that are a chance assemblage of clicks from different sources.

KERNO-F evaluates the ‘coherence’ of trains found by measuring the rates of change, through the train, of all the click features above and various derivatives, plus measures of the differences between successive inter-click intervals, and measures of the context of the clicks. Approximately 80 features and derivatives are used in the allocation of trains to the ‘quality’ classes, high, moderate and low that represent the confidence of the classification as a sequence that comes, at least in part, from a true train source. The quality and species classifications are orthogonal – i.e. the quality category of a train has no bearing on its species category and vice versa.

The KERNO-F species guild classification uses an ensemble of about 70 features and derivatives to construct distance metrics that are both specific to each pair of guilds (NBHF, other cetaceans, sonars and unclassified)  and are directional. This is because some features, such a click frequency, may conclusively indicate that a train is not an NBHF train, but cannot conclusively indicate that a train is an NBHF train.

The classifier includes a feedback process, run after the whole file has been processed, in which detections in each minute are potentially subject to re-classification based on the findings in an 11 minute long window centred on the focal window. The overall magnitude of changes made in the file are reported at the end of the process. The species classifier generates two levels confidence, with a large subset of true detections being ‘high confidence’ and the rest ‘low confidence’.

The KERNO-F classifier is about 6,000 lines of code, uses several hundred empirically determined numerical constants, about 2000 logical operators, nested up to 9 deep, and many recursive structures. Consequently it is not practical to validate it, either by reading the code or by analytical methods, which would necessarily be more complex than the primary code. This situation is common to many modern classifiers and makes extensive empirical testing, as in this study, essential.

Although the process itself cannot be directly validated some potential risk factors can be usefully predicted from the nature of the detection process. In particular:

1. Irregular trains are more likely to be missed.
2. Slower click trains are less likely to be detected particularly when the number of logged noise clicks is high.
3. The click rate assessment for slow click trains is more likely to be incorrect.
4. Trains of more distinctive clicks, e.g. NBHF clicks, can be identified at lower false positive levels than trains of less distinctive click, e.g. other cetaceans making short clicks.
5. During periods in which boat sonar features are present the classification of cetaceans will be inhibited to some degree.
6. Similarly a mixture of species groups may temporarily raise the detection threshold for each group.

*Error estimation:* The KERNO classifier used on C-POD data included a ‘self-assessment’ of potential errors based on the ratio of error sources to species found. This is not in a fully functional state in version 1.0 of KERNO-F, but does include assessment of the distribution of both click rates and click frequencies to enable reporting of anomalies.

*KERNO-F Settings*: Version 1.0 of KERNO-F is used throughout this study. Chelonia states that this version will be retained indefinitely without any minor or other improvements. Further classifier development will be in later versions.

Advanced settings for the KERNO-F classifier are provided in the F-POD software primarily for analysis of data sets with unusual sources of interference that need to be excluded by filtering within the train detection process. The default settings were used throughout in this study and are shown in Fig C.


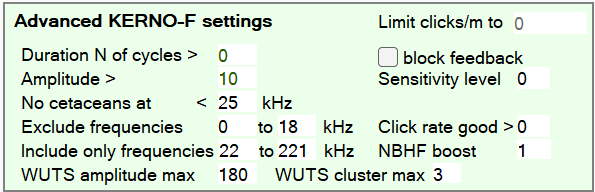


Fig C. The default advanced settings used in this study.

*Receiver Operating Characteristic curves:* ROC curves are not presented here because they are always context dependent. In contrast to the context being the Gaussian noise of radar receivers, which was the origin of this approach to describing the effect of varying detection thresholds, the context of F-PODs is the ambient soundscape, which is itself highly variable and difficult to characterise in terms that reflect its impact on the detection of trains. Where waves produce noise surges the ROC curve must move substantially to the right and back again several times each minute. Instead the standard thresholds that define Quality levels, High, Moderate, Low, have been chosen on the basis of their performance on data from very diverse data sets ranging from mangrove swamps to sites with moving sand or large waves. The use of High and Moderate quality combined with High species confidence is the selection recommended for general use by Chelonia and has been used in this study.

# Data structure

*Reading the FP1 file:* The source code for this can be downloaded from any copy of the FPOD app. As part of the process three features are derived ‘on the fly’ for each click, frequency, bandwidth and NBHF index.

*Click Frequency* *in kHz:*  The frequency of clicks stored by F-PODs cannot be derived from a Discrete Fourier Transform or similar wavelet transform as only a few data points are saved from the click. Wave peaks found in real time and used to measure the wavelengths (IPI, inter-peak-intervals) listed above in data capture.

The frequency of clicks with four or less cycles is based on the IPIs captured around the loudest wave in the click. In the case of clicks with only one IPI, which is in 250ns units there are steps in the possible frequency calculated that are 10kHz at 200kHz, 5kHz at 140kHz and 1kHz at approx. 60kHz.

For clicks with five or more cycles the frequency is based on the logged duration of the click, or the first 16 cycles, whichever is shorter as duration logging ends at the 16^th^ cycle. This frequency approaches the value that would be given by a DFT, except that the DFT effectively weights the loudest cycles in the click while the F-POD calculation described here does not.

*Click Bandwidth:* This metric corresponds conceptually to conventional measures of the bandwidth of the frequency spectrum but it is an arbitrary measure that is empirically based, and sums the ratio of changes in amplitude and IPI of the waves before and after the loudest cycle to that cycle. The shortest clicks do not have values for each of these and are forced to high bandwidth values while clicks with very little change in wavelength or amplitude around the peak get very low bandwidth values.

*Click NBHFindex:* This is an empirically based derivative which aims to represent the resemblance of a click to a canonical NBHF click. It does not determine any species classification on its own because its value is strongly affected by the amplitude of the click e.g. a very weak click from a porpoise will generally appear to have few cycles and a high bandwidth when much of the click is hidden in noise.

This index combines several logged features of a click to determine a value that seeks to represent how similar it is to a typical NBHF click. The process is based in empirical testing and uses a definition of a typical NBHF click that can be varied by the user, as shown in Fig D, to accommodate the variation found between NBHF clicks from different species or locations. The click features used contribute to a simple score:

| *Click feature* | *Assessed on:* |
| --- | --- |
| Frequency (kHz) | How close it is to the NBHF target frequency in Fig D. |
| Number of cycles | How close it is to the NBHF target number, with increasingly numbers of cycles above 50 over the target being increasingly penalised because they are more often made by boat sonars. |
| Wavenumber of loudest cycle (‘Pk@’) | Similar process to above |
| Number of amplitude reversals in the envelope of click peaks | Large numbers score negatively while low numbers on longer clicks score positively. |
| Range of wavelengths within the click (IPIs) | Low ranges in long clicks score high and conversely.  Also sets a low maximum score if high. |


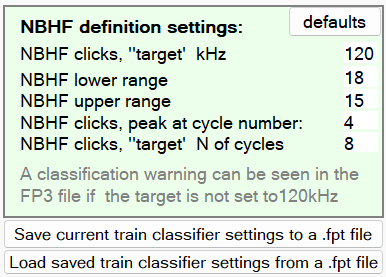


Fig D Target NBHF click description

*Reading the FP3 file:* The FP3 file is generated by the KERNO-F algorithm and contains records of only those clicks that were identified as belong to a train. Click records have the 16bytes of primary data, as in the FP1 file and another 16bytes holding various features of the train the click was in, so that filtering by those train features is possible. The train features are:

- Species guild
- Quality level (confidence that the train came from a train source)
- Species identification confidence, high or not. High confidence typically excludes 0-5% of trains that are closest to the decision level of the classifier.
- median pulse repetition frequency (PRF)
- click rate confidence on a scale of 0 -15 where the risk of error is lower for higher scores.
- median kHz
- average number of cycles in the clicks
- average amplitude
- average wavenumber of loudest wave in click
- average bandwidth
- estimated risk of coming from a weak unknown train source
- presence of a preceding gap in the train
- TrainID – this identifier increments from 1 to 255 which is the maximum number of trains that can be identified in one minute.

Also three additional click feature:

- *Cluster size* is a measure applied to clicks that are identified during processing as representative of the series of clicks that can follow when a click first reaches the logger and is then followed by one or more clicks that have been generated from the original click by reflection or refraction in the course of propagation. These collectively are a ‘multipath cluster’ and the size is the number of clicks it contains. See Fig E. The first click may sometimes be weaker than subsequent members because it may come from well off the acoustic axis of the dolphin’s sound production while the much louder on-axis sound arrives later after being reflected by the sea surface.
- *Cluster lowest frequency:* the lowest frequency found in the cluster, also:
- *Cluster highest frequency*


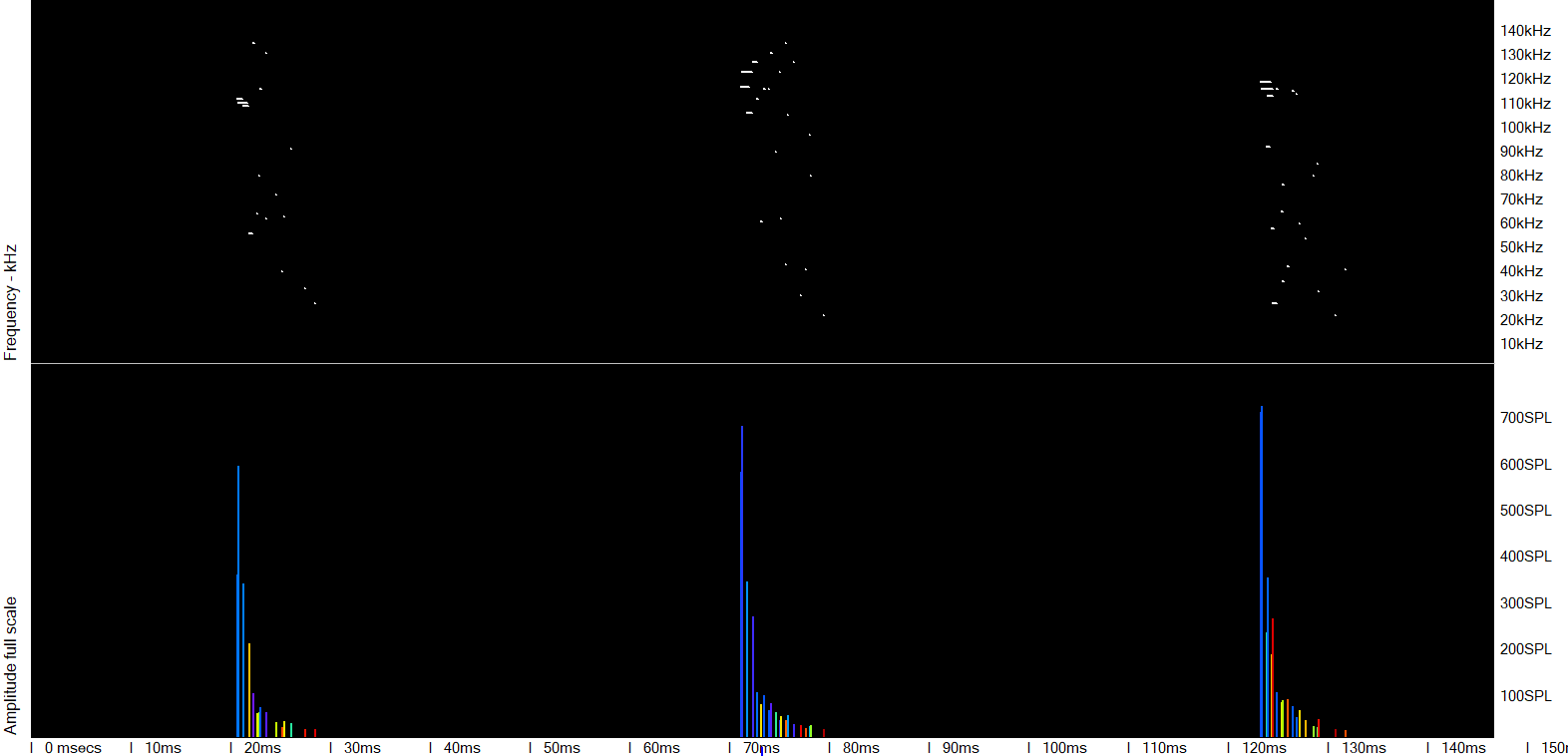


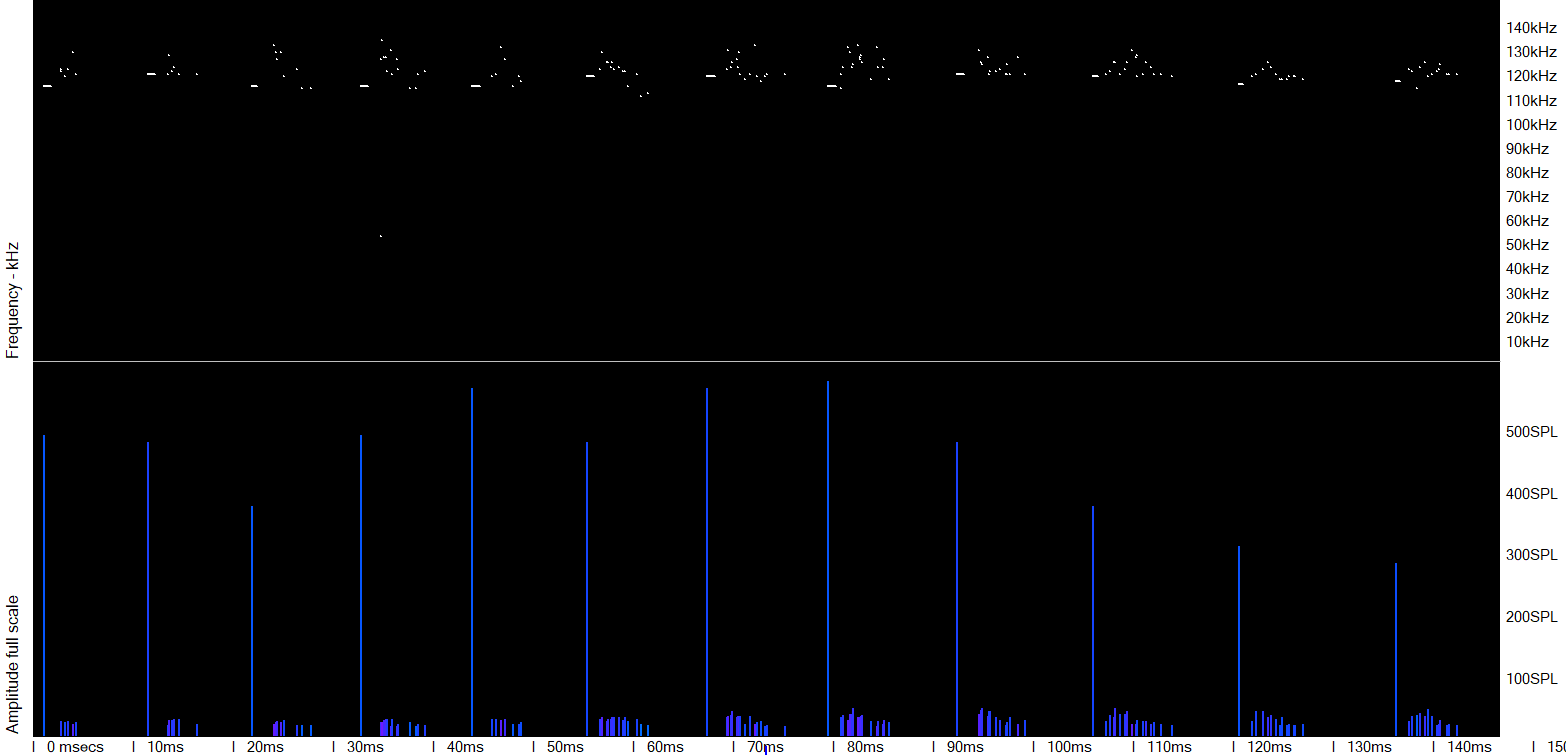


Fig E. Characteristics of multipath clusters from a dolphin in the upper two panels and from a porpoise in the lower two. The later arriving dolphin clicks reflect the different dominant frequencies in off-axis regions of the sound beam of the dolphin, with some modification due to frequency dependent absorption increasingly removing the higher frequencies. The later arriving porpoise clicks reflect the much greater frequency coherence of the sound beam of the porpoise, but the first arriving loud click here is generally lower in frequency. This is not typical and could arise from the direct path having been off-axis with the slightly higher axial frequencies appearing in echoes from the sea surface.

# BlackCeTrends Data files

The file name structure is:

Country 1 Character – first letter in name

Site Name

Site Number 01, 02 etc if logger position has moved by > 200m

Date of start yyyy mm dd

FPOD number

File number – the data from one deployment may be split into several files

Part size – the duration of the file after cropping
